# Accidental intra-thecal tranexamic acid injection during spinal anesthesia for myomectomy: Case report and review

**DOI:** 10.1016/j.amsu.2022.104646

**Published:** 2022-09-14

**Authors:** Khalid Abbas Owish Suker, Muna Omer Mohamed Elatta, Mohmmed Mahmmoud Fadelallah Eljack, Abdelrahman H. Abdelmoneim, Hiba Awadelkareem Osman Fadl, Ghazi Gasmalla Mohamed

**Affiliations:** a Department of Physiology & Anaesthesia, Faculty of Medicine, University of Bakht Alruda, Aldueim, Sudan; b College of Health Sciences, Gulf Medical University, Ajman, United Arab Emirates; c Obstetrician & Gynaecologist, Bahri Teaching Hospital, Khartoum, Sudan; d Faculty of Medicine, University of Bakht Alruda, Aldueim, Sudan; e Clinical Immunology Resident, Sudan Medical Specialization Board, Khartoum, Sudan; f Faculty of Medicine, Al-Neelain University, Khartoum, Sudan; g Department of Haematology, Faculty of Medical Laboratory Sciences, Al-Neelain University, Khartoum, Sudan; h Department of Medical Laboratory, Sudanese Medical Research Association, Khartoum, Sudan; i Gulf Medical University, College of Health Sciences, Anesthesia Technology Department, Ajman, United Arab Emirates

**Keywords:** Tranexamic acid, Spinal anesthesia, Operations, Complications

## Abstract

**Introduction:**

Inadvertent drug administration stays one of the reasons for avoidable morbidity and mortality complications around the globe. This report will talk about a case of inadvertent Intra-thecal Tranexamic Acid injection for a myomectomy operation, which ultimately leads to potential complications for patients.

**Case presentation:**

A 33-year-old HIV-positive woman with dysmenorrhea for two years was diagnosed with uterine fibroids and scheduled for a myomectomy. After spinal anesthesia, the patient developed nonresponsive myoclonic seizures, so she was sedated, intubated, and hooked up to a mechanical ventilator. However, her condition continued to deteriorate, and she developed narrow complex tachycardia, which was controlled but later developed systole and she died. A second check for anesthetic drugs revealed that she was given tranexamic acid rather than bupivacaine.

**Discussion:**

a succinct review was done to analyze the available current published data in the PubMed database about the problem, which concludes that it was described more often in developing countries. This is due to low obedience to medication safety procedures when managing drugs in operation rooms; however, more data is required to validate this assumption. Tranexamic acid is usually safe however improper use may cause catastrophic Gastrointestinal, cardiac and neurological complications.

**Conclusion:**

Suggestions are put together for the local drug enterprises to modify the appearance of the drug ampoules to reduce the possibility of similar incidents in the time to come. Implement drug preparation systems and double-check the medications before administration by more than one anesthesia staff.

## Highlights

• Inadvertent drug administration stays one of the reasons for avoidable morbidity and mortality complications.
• Little is reported about the effect of intrathecal administration of TXA, particularly in Sudan.
• Tranxemic acid is safe drug, However improper use may lead to fatal complications.
• Most of the reported cases ended in death despite extensive management.
• This case reports demonstrating a call for implementing some changes to prevent the recurrence of these accidents in the time to come.

## 1 Introduction

Drug side effects exist as a main cause of injury as well as mortality across the globe [1]. Practices such as lookalike medications put together, improper labeling, the location of ampules and syringes, the non-existence of a drug double-check system, anaesthesiologists’ distraction or negligence, poor communication, and exhaustion. All these exercises are considered potential causes of medical errors [[1], [2], [3]].

Tranexamic acid (TXA), a lysine analog that acts as an antifibrinolytic by interacting with plasmin and plasminogen in a dose-dependent manner, is commonly used during surgical procedures to manage to bleed. It has been related to earlier intrathecal injections that caused severe back pain, lower limb myoclonus, and agitation developing into a general seizure, ventricular arrhythmias, and death in some individuals [4,5].

In our case, we report. A 33-year-0 has been injected with tranexamic acid instead of hyperbaric bupivacaine for spinal anesthesia due to a mix-up between two separate ampules with similar looks, resulting in seizures, polymyoclonus, cardiac arrhythmia, and death. This case report is in line with the SCARE 2020 criteria [6].

## 2 Case presentation

A 33-year-old woman with HIV who lived on treatment for 10 years, presented to an obstetrics and gynecology clinic with dysmenorrhea for 2 years. Clinical examination and abdominal ultrasonography confirmed the diagnosis of uterine fibroids, and the patient was scheduled for a myomectomy by the obstetrician.

The patient's Investigations showed: Haemoglobin of 10.4 g/dl, Platelets: of 353 × 103/μL, and Serum Na+ 142 mmol/L, K+ 4.0 mmol/L.

The patient was classified as ASA class 1 by the anesthetic team during pre-operative preparations, the patient was prepared for spinal anesthesia which was confirmed at the point of L3 and L4 with 4 ml of Bupivacaine injected when CSF dropped, and then the patient had been put to supine on the operating table. After 2–3 minutes of spinal technique, the patient complained of accessive sweating and severe back and leg pain, which led her to cry, as well as rigor and a rise in blood pressure to 180/100 mmHg.A few minutes later, the patient developed focal seizures, which progressed to myoclonic seizures. The patient was given 100 mg of fentanyl to relieve the pain, as well as 15 mg of Midazolam but her condition continued to deteriorate, and she was rushed to the high dependency unit (HDU), intubated, connected to mechanical ventilation, and given Atracurum 40 mg, Medizolam by syringe pump rate 5mg/hour as sedative and Propofol 10mg/hour. The patient then developed narrow complex tachycardia with a heart rate of 180 beats per minute, BP 180/120 managed by an Emergency room (ER) physician using carotid massage with heart rate declined to 120, and a Labetalol injection of 20 mg was slowly administered.

The anesthetist determined that the problem could be due to hypersensitivity to bupivacaine firstly before complications developed and placed the patient on intralipid infusion, as well as performed A double-check of anesthetics was performed after the onset of the complications, and an anesthesia technician performed a check and discovered a broken tranexamic acid ampule in the anesthetic tray, which like bupivacaine, necessitated changing the plan with intralipid stopped and sedation maintained, but the patients developed excessive secretions from the endotracheal tube, necessitating endotracheal suction, then suddenly patient developed asystole and CPR was done for 30 minutes but unfortunately, the patient passed away. There was no CT scan for scanning the spinal cord at our facility.

## 3 Discussion

Little is reported about the effect of intrathecal administration of TXA, particularly in Sudan [7].

TXA is one of the antifibrinolytic medications that function as a plasmin inhibitor by binding with plasminogen through their lysine receptor sites, averting fibrin degradation [2,3].

Furthermore, TXA is commonly used in various surgeries belonging to obstetrics, gynecology, and cardiology [8,9] Medication errors are one of the most frequent medical flaws [10] It may occur due to many reasons for example improper labeling of the syringe or filling, carelessness, fatigue of healthcare providers [11].

Tranexamic acid is usually a safe, well-tolerated, and effective method to prevent abnormal bleeding in different patient groups [4]. However, when used in overdoses or incorrectly, it could have catastrophic complications [4].

TXA was recorded to cause adverse effects. Most of them are including GIT involvement, e.g., nausea, vomiting, and diarrhea. Other side effects are life-threatening and could be cardiac, and neurological related [4].(5).

The present case showed that a patient with HIV presented with dysmenorrhea for several months and has been diagnosed with uterine fibroids by Ultra-sonography of the pelvis.

During a myomectomy procedure, TXA was administrated to her intra-thically by error, and this caused immediate back pain, complicated neurotoxicity, focal seizures, which progressed to myoclonic and seizures. The patient was then intubated, and connected to mechanical ventilation to manage, all necessary medications are given, but all failed. The patient passed away from neurotoxicity and high blood pressure, cardiac arrest, and respiratory failure.

The different complications related to the use of intrathecal tranexamic acid could be due to the different doses used. The higher dose in this report (400 mg) is like in earlier reports, explaining the poor prognosis. While the report of the lower dose was associated with more favourable outcomes [4].

**Fig. 1:**
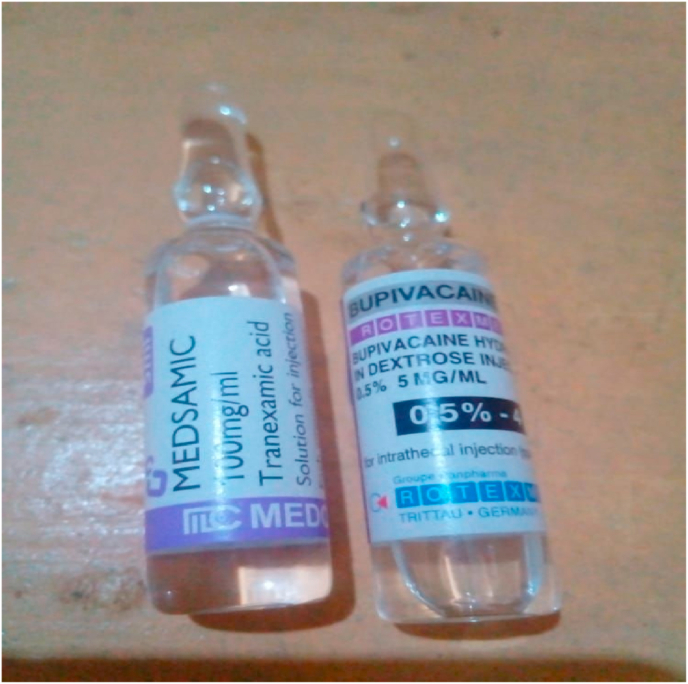
two ampules of bupivacaine and tranxemic acid.

The first case was reported by Wong et al., in 1988 in an 18-year-old appendectomy patient who luckily survived the incident without major complications [5]. There were several other reports over the years. Despite some companies changing the shape of the ampules after earlier reports in 2011 [1], there are still others that still use the closely matched ampules, which could explain why mistakes keep happening over the years as clear in Fig. 1.

Several reviews were done before, on this topic, the latest was in 2019 [7], which approaches the problem by analyzing the human factor involved in it. Here we focus more on the recently published evidence about the problem (Table 1).

**Table 1:** summary of retrieved articles.

author	dose	Operation	Sign and symptoms	management	outcome
Mahmood et al., [7]2012	300 mg	Skin grafting	severe pain in back and gluteal region, Hypertension	Fentanyl, midazolam, propofol, lidocaine	Full recovery
Srivastata et al. [8] 2012	350 mg	cholecystectomy	Severe pain in back and legs, generalized convulsions	Midazolam, fentanyl, diazepam, adrenaline, lidocaine	Death
Antwi-Kusi et al. [9] 2013	NA	Caesarean section	convulsions, No sensory or motor block. Hypertension,	General anesthesia, anticonvulsants, thiopental.	Death
Goyal w al [4].2014	250 mg	Inguinal Hernia Repair	Back and leg pain, convulsion	Fentanyl, midazolam, lorazepam, phenytoin, propofol,	Full recovery
Hatch et al. [10] 2016	200 mg	Caesarean section	No neurological block, tetany, and hypertension	Anticonvulsant dexamethasone,	death
ElKhateeb et al. [11] 2017	160 mg	Caesarean section	Generalized convulsion, arrhythmia and sever back pain	General anesthesia. Anticonvulsant CPR	death

Regarding the presentation, it was almost similar in most of the cases, with the condition beginning with failure to introduce a sensory or a motor block, followed by a sensation of severe back and leg pain, and ending with convulsions or arrhythmia in most of the cases.

the similar presentation also explains the similar management plans between the reports focusing on general anesthesia and anticonvulsant followed by rescission and ICU admission in most of the cases. Some records also report the use of dexamethasone like in Hatch et al. study [11].

Unfortunately, most of the reported cases ended in death despite extensive management [[9], [12]] This is further complicated by the difficult access to advance medical equipment in developing countries as shown by the difficulty of gain access to CT in our case.

Likewise, there are many sporadic clinical cases are reported worldwide, in which intrathecal TXA injection was administered instead of hyperbaric bupivacaine for spinal anesthesia [1].

this recurrence of similar medical errors worldwide showed that it might result from a shared root, so the similarity of the shape of the two ampules is the most probable cause.

Earlier solutions before blocking include ensuring that it is the correct patient, agent, and site. Cross-checking the drugs with the operating department practitioner/anesthetic assistant may help.

We recommend that anesthetists, anesthesia technicians, nurses, pharmacists, and other health care providers form a group to discuss the prevention and management of such unintentional cases. For such errors, hospitals should establish strong supervision and quality control system.

Manufacturer to unique distinct ampoules in shape and color with noticeably clear labeling enabling everyone to read, all critical medication anesthesia, particularly TXA as it is known to be the highest reports of accidental administration medical flaws. Also, medication errors could be avoided by reducing staff tiredness to a minimum.

Hospitals should improve the well-organized system for storage inside pharmacies and transportation of TXA to the theatre, this system should be implanted strictly.

This case could be the precursor to a quality improvement project incorporating changes associated with patient safety.

## 4 Conclusion

This case report shows the substantial complication associated with accidental intrathecal injection of tranexamic acid during surgical procedures, which calls for implementing some changes to prevent the recurrence of these accidents in the time to come.

## Ethical approval

Ethical approval to publish this case was obtained from research and Ethics committee, Bahri Teaching Hospital, Khartoum, Sudan.

## Sources of funding

None

## Author contribution

All Authors Contributed equally in this work.

## Registration of research studies

1. Name of the registry:

2. Unique Identifying number or registration ID:

3. Hyperlink to your specific registration (must be publicly accessible and will be checked):

## Guarantor

Dr. Mohammed Mahmmoud Fadelallah EljackTeaching assistant, Faculty of Medicine and Health Sciences, University of Bakht Alruda, Ad Duwaym Sudan.

## Consent

Written informed consent was obtained from the patient for publication of this case report and accompanying images. A copy of the written consent is available for review by the Editor-in-Chief of this journal on request.

## Provenance and peer review

Externally peer reviewed not commissioned.

## Declaration of competing interest

No conflict of interest.
